# Evaluation of Early Outcomes of Trapeziectomy With Suture-Button Suspensionplasty and Palmaris Longus Zig-Zag Augmentation for Thumb Carpometacarpal Osteoarthritis: A Prospective Case Series

**DOI:** 10.7759/cureus.117885

**Published:** 2026-10-13

**Authors:** Malkesh Shah, Chirag Kapoor, Prateek Randhawa, Devanshu Garg, Harsh S, Sankalp S Jain, Aadesh Gandhi

**Affiliations:** 1 Orthopaedics, Sumandeep Vidyapeeth Deemed to be University, Vadodara, IND; 2 Orthopaedics, Smt. B. K. Shah Medical Institute & Research Centre, Sumandeep Vidyapeeth Deemed to be University, Vadodara, IND; 3 Orthopaedic Surgery, Smt. B. K. Shah Medical Institute & Research Centre, Vadodara, IND; 4 Orthopaedics, Dhiraj Hospital, Smt. B. K. Shah Medical Institute &amp; Research Centre, Sumandeep Vidyapeeth Deemed to be University, Vadodara, IND; 5 Orthopaedics, Dhiraj Hospital, Sumandeep Vidyapeeth Deemed to be University, Vadodara, IND

**Keywords:** carpometacarpal joint, grip strength, hand surgery, palmaris longus, pinch strength, suture-button suspensionplasty, thumb cmc arthritis, thumb osteoarthritis, trapezial space ratio, trapeziectomy

## Abstract

Aim

The study aimed to evaluate the early (two-month) clinical, functional, and radiographic outcomes of trapeziectomy with suture-button suspensionplasty (SBS) augmented by a palmaris longus (PL) zig-zag ligament reconstruction in thumb carpometacarpal osteoarthritis.

Objectives

The objectives of the study were: to measure change in pain, grip strength, pinch strength, and thumb range of motion; to assess functional recovery using the QuickDASH score; and to determine maintenance of the trapezial space ratio at two months after surgery.

Background

Thumb carpometacarpal (CMC) osteoarthritis significantly impairs hand function due to biomechanical demands placed on the trapeziometacarpal joint. Trapeziectomy with suture-button suspensionplasty (SBS) provides suspensory fixation of the first metacarpal to the second, conferring immediate stability that permits early mobilisation; in this series, the construct was augmented with a palmaris longus graft.

Methods

This was a pilot prospective case series. Ten patients (age 50-75 years) with Eaton-Littler stage III-IV disease underwent SBS using a double endo-button adjustable loop with palmaris longus graft zig-zag augmentation. Follow-up duration was 2 months. Outcomes included pain visual analogue scale (VAS), grip strength, pinch strength, range of motion (ROM), and trapezial space ratio (TSR). Statistical analysis was performed using the Wilcoxon signed-rank test, with two-tailed asymptotic p-values and effect sizes calculated as $r = \frac{Z}{\sqrt{N}}$ (N = 10).

Results

Visual analog scale improved from 8.1 to 2.2 (p = 0.005). Grip strength improved from 54% to 74% (p = 0.007). Pinch strength increased from 48% to 74% (p = 0.007). Palmar and radial abduction improved by 22° and 15°, respectively. TSR was maintained at 84%. Complications comprised one transient complex regional pain syndrome and one superficial radial nerve neurapraxia; both resolved completely with conservative management. There were no infections, hardware failures, or button cut-outs.

Conclusion

In this small, single-arm series, trapeziectomy with suture-button suspensionplasty and palmaris longus zig-zag augmentation was associated with improvements in pain, function, strength, range of motion, and radiographic trapezial height at two months. In the absence of a comparison group, these findings are descriptive and hypothesis-generating; the two-month endpoint cannot address durability of the clinical result, late implant-related complications, or long-term maintenance of trapezial height.

## Introduction

Thumb carpometacarpal (CMC) osteoarthritis is the most common symptomatic arthritis of the hand, with an age-adjusted radiographic prevalence of approximately 7% in men and 15% in women, rising to 17-33% among postmenopausal women, and it accounts for a steadily increasing surgical caseload [1-3]. The trapeziometacarpal joint is a biconcave saddle joint exposed to high biomechanical loads - a 1-kg pinch generates roughly 13 kg of compression - which, together with progressive ligamentous degeneration, predisposes to instability and functional decline [4,5]. Numerous surgical options exist for advanced CMC arthritis, including simple trapeziectomy, ligament reconstruction tendon interposition (LRTI), abductor pollicis longus (APL) suspensionplasty, hematoma distraction arthroplasty, arthrodesis, arthroplasty, and various suture-based suspension techniques [6-11]. No single procedure has proven universally superior across all outcome domains [12]. Although trapeziectomy reliably reduces pain, proximal migration of the first metacarpal and resultant instability are well documented [13].

Suture-button suspensionplasty (SBS) was described as a means of preventing proximal metacarpal migration under pinch loading while avoiding the morbidity of tendon harvest or K-wire immobilisation. SBS uses a cortical button-to-button construct to suspend the first metacarpal from the second. The construct is designed to provide immediate mechanical stability, and the published series employing it have permitted early rehabilitation [14,15]. Multiple mid- and long-term series report sustained trapezial height preservation, reduced subsidence, and improved grip and pinch strength compared with conventional ligament reconstruction or trapeziectomy alone, with low complication rates during follow-up periods of up to 14 years [16-20].

On this basis, we conducted a pilot prospective case series (n = 10) of trapeziectomy followed by SBS augmented with a palmaris longus (PL) zig-zag sling. The PL augmentation is intended to provide an additional volar soft-tissue restraint that supplements the suture-button construct without added donor-site morbidity. This study reports early clinical (range of motion, strength, VAS, QuickDASH), radiographic (trapezial space ratio), and safety outcomes following this combined suspensory and volar augmentation technique.

## Materials and methods

Study design

A prospective observational case series was conducted over 10 months, with postoperative follow-up completed at 2 months. The study was approved by the Institutional Ethics Committee of SBKS Medical Institute & Research Centre, Sumandeep Vidyapeeth (Deemed to be University) (approval number SVIEC/ON/MEDI/RP/AUG/25/3, dated 14/08/2025) and was conducted in accordance with the Declaration of Helsinki and with institutional ethical guidelines. Written informed consent for participation, for the surgical procedure including the palmaris longus augmentation, and for the use of clinical photographs and radiographs for publication, was obtained from every patient.

A two-month endpoint was selected a priori because it corresponds to completion of the standardised rehabilitation protocol and to the earliest point at which the accelerated-recovery advantage attributed to suture-button suspensionplasty can be assessed. This is therefore a short-term assessment; it cannot address durability of the clinical result, late implant-related complications or long-term maintenance of the trapezial height.

Participants, eligibility, and sample size

Patients were considered eligible for inclusion if they were between 50 and 75 years of age, had Eaton-Littler stage III or IV trapeziometacarpal osteoarthritis on radiographic staging [21], had persistent symptoms despite at least six months of conservative treatment, had a clinically demonstrable palmaris longus tendon available for augmentation, and were able to comply with the postoperative rehabilitation protocol. Patients were excluded if they had an active local or systemic infection, advanced scaphotrapeziotrapezoid arthritis, severe metacarpophalangeal hyperextension greater than 30 degrees, any previous surgery on the thumb carpometacarpal joint, or a peripheral neuropathy impairing hand function.

Ten patients diagnosed with Eaton-Littler stage III-IV thumb CMC osteoarthritis were included, fulfilling the inclusion and exclusion criteria. No a priori sample size calculation was performed. As a pilot case series evaluating a technical modification, the study recruited all consecutive eligible patients presenting during the 10-month study period (convenience sampling). The resulting cohort of 10 patients is not powered for comparative inference; a post hoc review confirmed that, for the within-subject effect sizes observed (r = 0.79-0.89), a sample of 10 paired observations was sufficient to detect the reported changes at α = 0.05, but the series remains exploratory and hypothesis-generating.

Outcome measures

The primary outcome measures were pain assessed on a 10-point visual analogue scale (VAS), grip strength and pinch strength expressed as a percentage of the contralateral side, and the radiographic trapezial space ratio (TSR). Secondary outcome measures were thumb range of motion, recorded as palmar and radial abduction, and the occurrence of complications. Patient-reported function was assessed with the shortened Disabilities of the Arm, Shoulder and Hand questionnaire (QuickDASH), originally developed and validated by Beaton et al. [22]. All outcome measures were recorded preoperatively and at two months after surgery by an assessor who was not the operating surgeon.

Radiological assessment

Standardised anteroposterior and oblique radiographs of the hand were obtained preoperatively in all patients, and magnetic resonance imaging was performed preoperatively in all 10 patients to confirm the stage of disease and to exclude scaphotrapeziotrapezoid involvement (Figure 1A-1D). Identical anteroposterior and oblique radiographic projections, obtained with the same positioning and technique as the preoperative films, were repeated at two months (Figure 2A, 2B). Magnetic resonance imaging was performed preoperatively only and was not repeated postoperatively.

**Figure 1:**
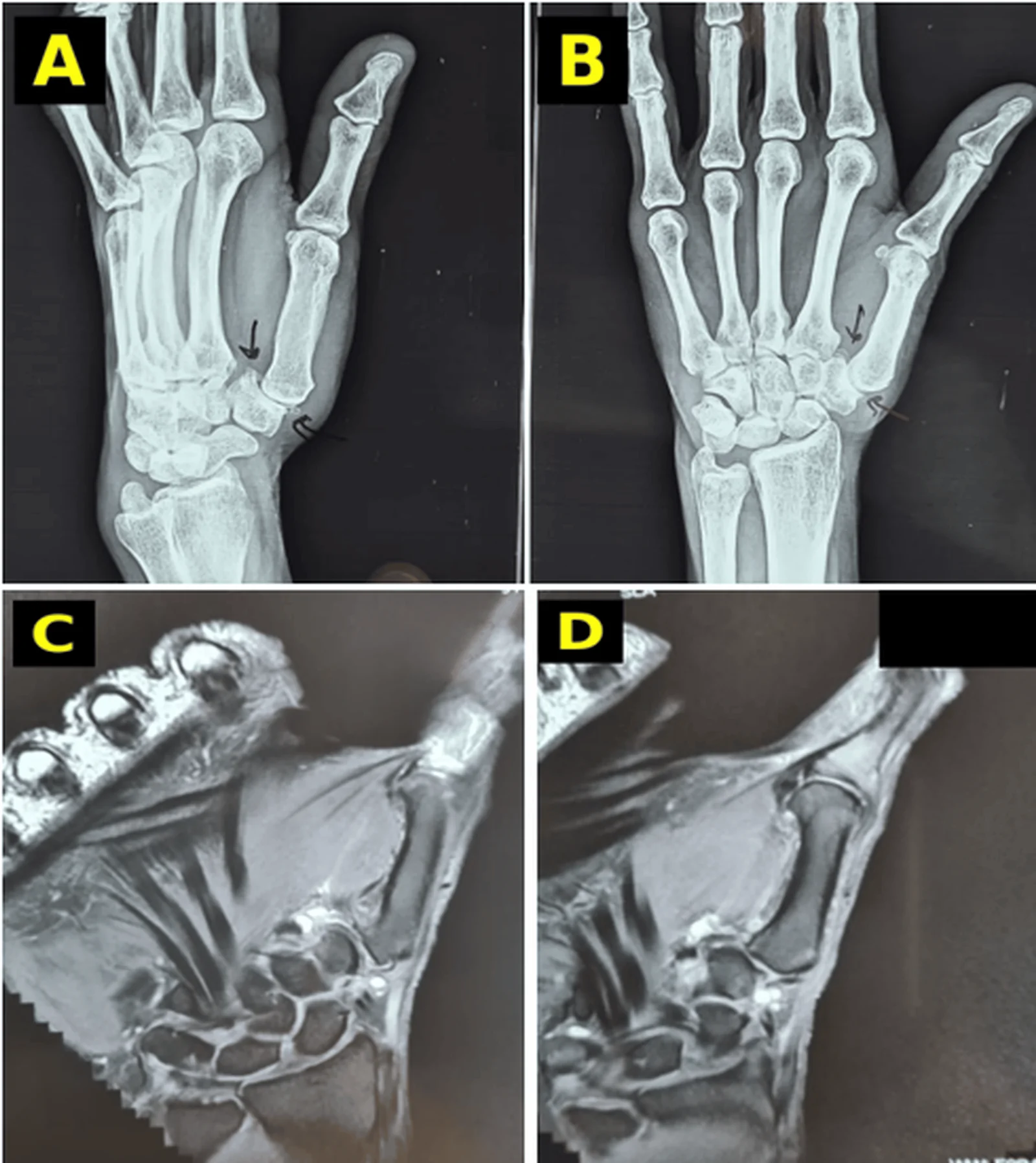
Preoperative radiological assessment. (A) Anteroposterior and (B) oblique radiographs of the hand demonstrating Eaton–Littler stage III–IV trapeziometacarpal osteoarthritis; arrows indicate loss of the trapeziometacarpal joint space and radial subluxation of the first metacarpal base. (C) Coronal and (D) sagittal magnetic resonance images of the same hand confirming trapeziometacarpal degeneration and excluding scaphotrapeziotrapezoid involvement.

**Figure 2:**
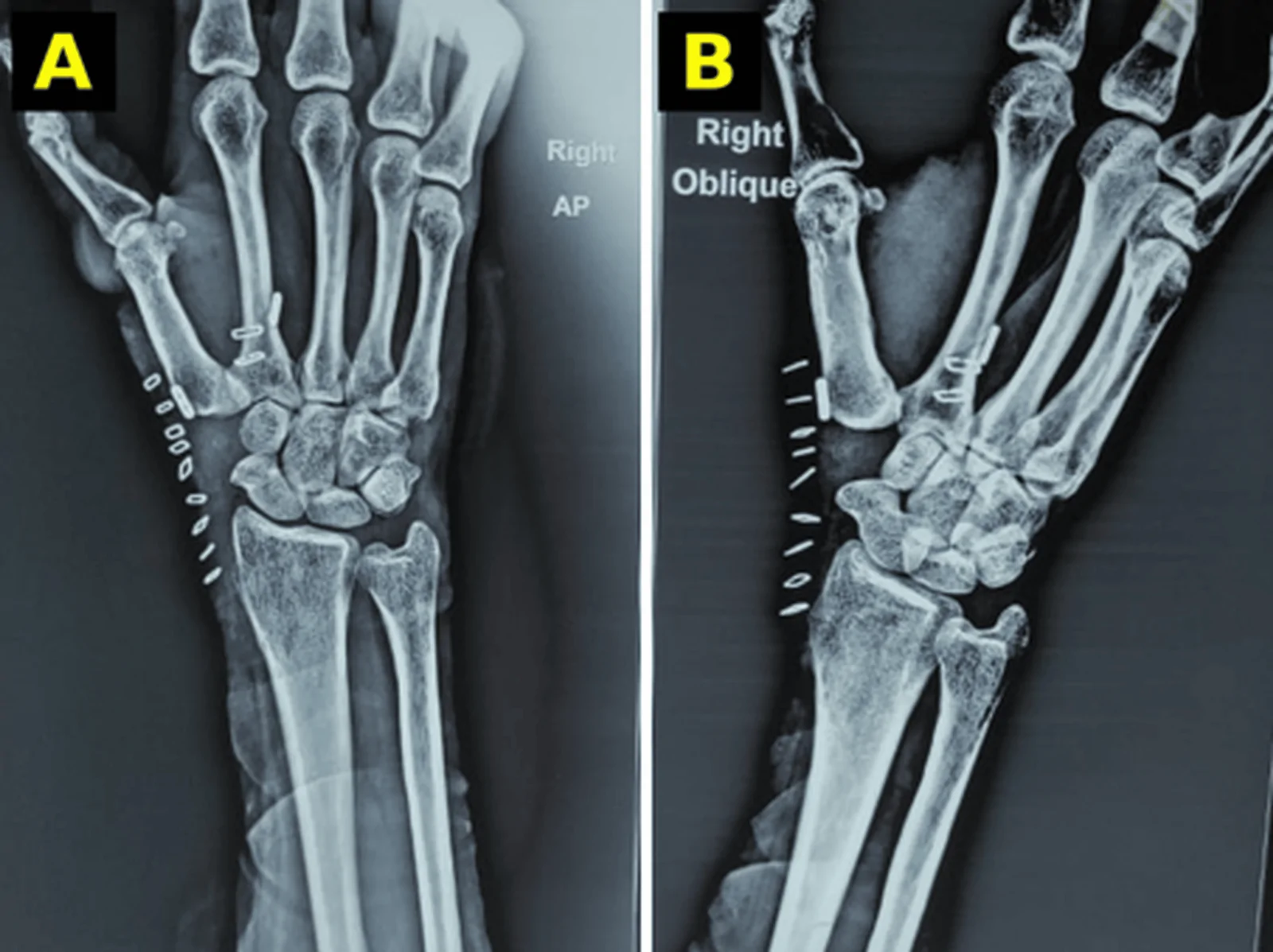
Postoperative radiographs of the hand at two months. (A) Anteroposterior and (B) oblique views, both demonstrating a maintained trapezial space and correct positioning of both buttons of the suture-button suspensionplasty construct.

The trapezial space ratio was calculated as the distance between the base of the first metacarpal and the distal pole of the scaphoid, divided by the length of the proximal phalanx of the thumb on the same radiograph, and was expressed as a percentage of the immediate postoperative value. All radiographic measurements were made independently by two observers who were blinded to each other’s readings, and the mean of the two values was used for analysis. Interobserver agreement for the trapezial space ratio was assessed using the intraclass correlation coefficient (two-way random-effects model, absolute agreement, single measures), which was 0.94 (95% CI 0.85 to 0.98), indicating excellent agreement.

Statistical analysis

Given the small sample size (n = 10), non-parametric methods were used throughout. The Wilcoxon signed-rank test was applied to compare preoperative and postoperative values for each outcome measure. For every comparison, the test statistic (Z), the two-tailed asymptotic p-value, and the effect size, calculated as $r = \frac{Z}{\sqrt{N}}$, where N is the number of participants contributing paired preoperative and postoperative observations (N = 10 for every comparison), are reported. A p-value below 0.05 was considered statistically significant. Interobserver agreement for radiographic measurements was assessed using the intraclass correlation coefficient. Continuous outcomes are reported as median (interquartile range) as the primary descriptive statistic, with mean ± standard deviation given alongside for comparability with the published literature. Analyses were performed using IBM SPSS Statistics version 26 (IBM Corp., Armonk, NY, USA).

Surgical technique

The procedure combined standard suture-button suspensionplasty (SBS) principles described by DeGeorge [14] and Yao [15,16] with a palmaris longus (PL) zig-zag augmentation developed for enhanced volar stabilisation. All procedures were performed by the same senior hand surgeon under regional anaesthesia (supraclavicular brachial plexus block). The patient was positioned supine with the operative limb on a radiolucent hand table, and a pneumatic arm tourniquet was inflated to 250 mmHg after exsanguination. Standard sterile preparation and draping were performed.

The palmaris longus zig-zag configuration described here is a modification of established ligament reconstruction principles rather than a new operation. Its use was reviewed and approved by the Institutional Ethics Committee as part of the study protocol. Every patient was counselled specifically that the augmentation is an addition to a standard trapeziectomy and suture-button suspensionplasty, that its incremental benefit is unproven, and that it requires harvest of the palmaris longus; written informed consent was obtained on that basis. The augmentation requires no additional incision beyond the standard palmaris longus harvest, adds approximately 15-20 minutes to the procedure, and does not alter the salvage options, should revision be required.

A 3-4 cm longitudinal dorsoradial incision was made over the first carpometacarpal (CMC) joint. Dissection was carried down carefully, protecting the superficial branches of the radial nerve and cephalic vein tributaries. A longitudinal capsulotomy was performed to expose the trapeziometacarpal joint (Figure 3A, 3B).

**Figure 3:**
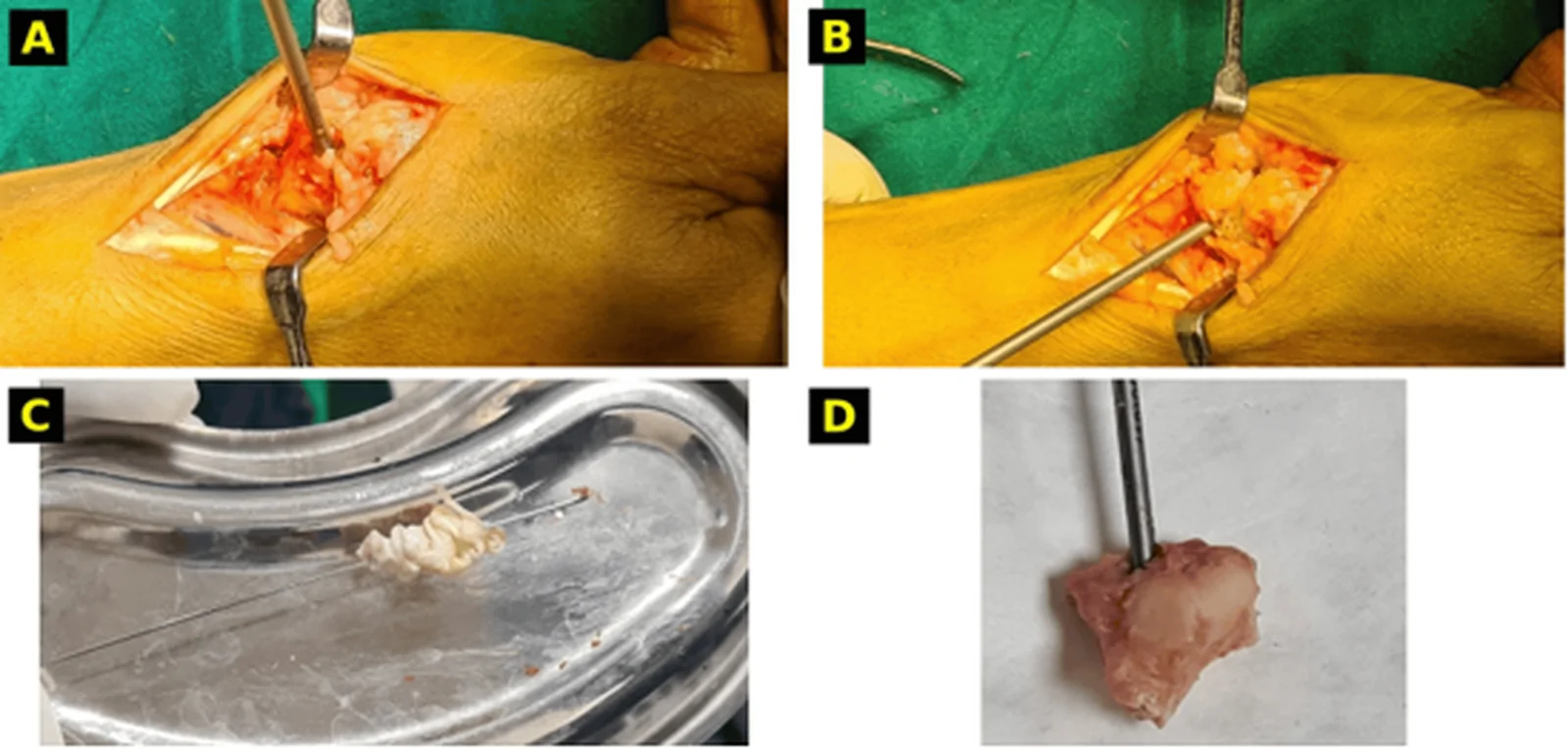
Intraoperative photographs. (A) Dorsoradial approach to the first carpometacarpal joint with the incision retracted. (B) Exposure of the trapeziometacarpal joint following longitudinal capsulotomy. (C) Excised trapezium in the instrument tray. (D) Close-up of the excised trapezium confirming complete trapeziectomy. Written consent for publication of clinical photographs was obtained from every patient.

Under fluoroscopic guidance, a 1.6 mm guidewire was inserted from the base of the first metacarpal towards the second metacarpal in the coronal plane, approximately 1 cm distal to the articular surface of the first metacarpal base. After confirming trajectory on anteroposterior and lateral fluoroscopic images, a 3.2 mm cannulated drill was used over the guidewire to create a bicortical tunnel through both metacarpals (Figure 4A-4C).

**Figure 4:**
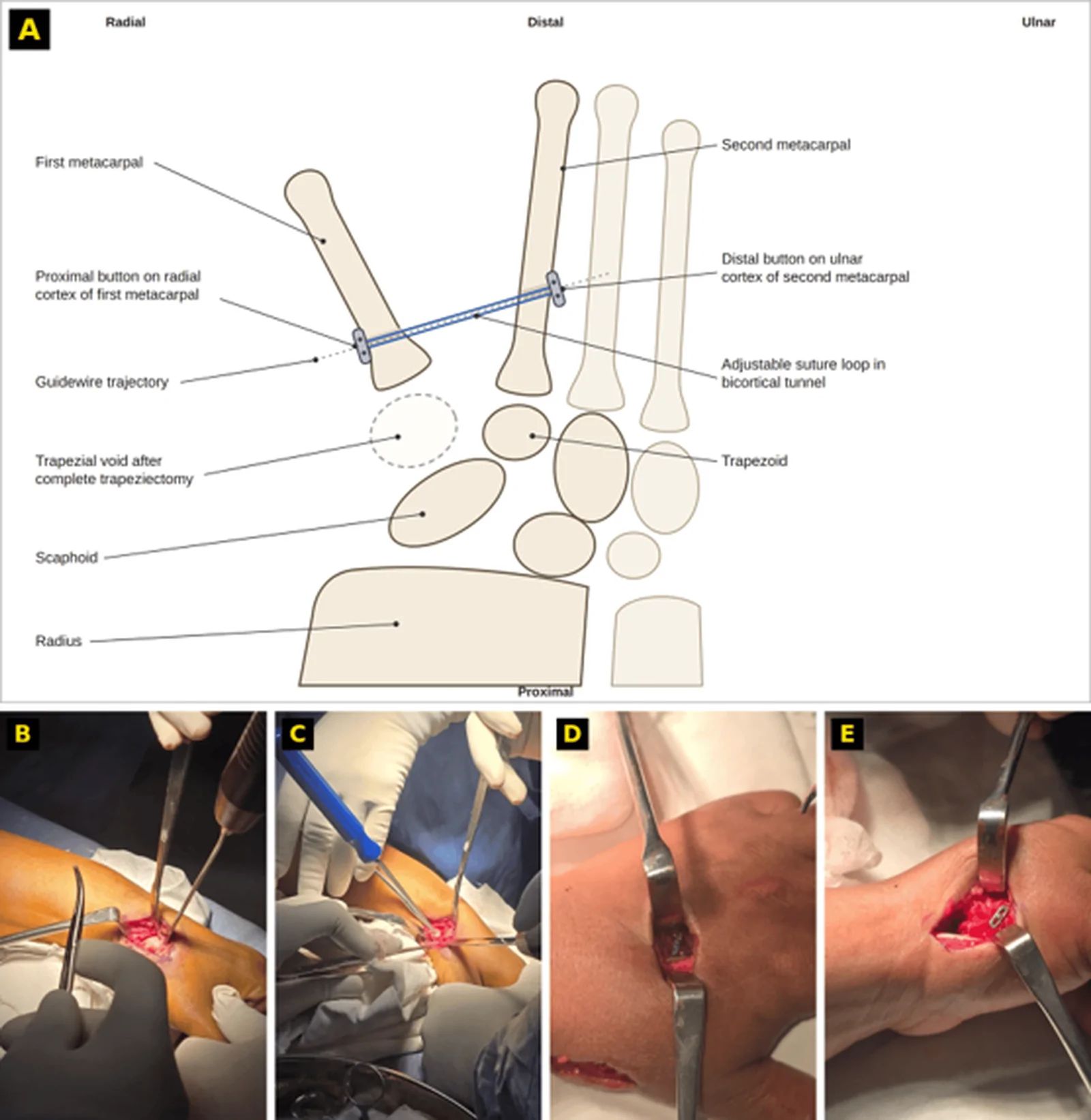
Schematic line diagram and intraoperative images of the suture-button construct. (A) Coronal schematic showing the guidewire trajectory, the bicortical tunnel through the first and second metacarpals, the trapezial void following excision, the distal button on the ulnar cortex of the second metacarpal, the proximal button on the radial cortex of the first metacarpal, and the adjustable loop. (B) Intraoperative photograph of the 1.6 mm guidewire in situ. (C) The 3.2 mm cannulated drill passing across both metacarpals. (D) The adjustable-loop device shuttled through the tunnel. (E) The deployed construct with the trapezial space restored. The schematic line diagram (A) was drawn as a vector illustration with the assistance of Claude (Anthropic, San Francisco, USA). The authors then reviewed and corrected it for anatomical and technical accuracy.

A single adjustable-loop cortical suspension device (Mini TightRope, Arthrex, Naples, FL, USA) with a 6 × 2 mm oblong cortical button at each end and a No. 2 braided non-absorbable suture loop was then passed through the tunnel. The distal button was deployed against the ulnar cortex of the second metacarpal, while the proximal button rested on the radial cortex of the first metacarpal (Figure 4D, 4E). The adjustable loop was tensioned incrementally under fluoroscopic control until the base of the first metacarpal was restored to the level of the base of the second metacarpal and a trapezial space of approximately 1 cm was re-established. Tensioning was stopped at that point; the thumb was then taken passively through full palmar abduction, radial abduction and opposition to the base of the little finger to confirm that motion was unrestricted, and a lateral pinch was simulated under fluoroscopy to confirm that the first metacarpal did not subside.

Complete trapeziectomy was carried out using rongeurs and osteotomes, following established trapeziectomy and suture-button suspensionplasty (SBS) techniques [14,18]. Volar ligamentous structures, the abductor pollicis longus (APL) subsheath, and sufficient capsular tissue were preserved for reconstruction (Figure 3C, 3D).

The palmaris longus (PL) tendon was harvested through a small transverse incision at the distal forearm. The tendon was isolated on a right-angled clamp, released proximally, and harvested in full length using a tendon stripper; the graft measured 12-15 cm in every case.

To augment volar stability, a zig-zag PL ligament reconstruction was performed (Figure 5). Given the critical role of the volar beak ligament in CMC stability [5], the PL tendon was passed beneath the APL subsheath to create a volar-to-dorsal sling. Capsular slits were created with a No. 15 blade, and the tendon was then woven in a zig-zag fashion through these slits, looped around the flexor carpi radialis (FCR) insertion, and crossed volarly beneath the base of the first metacarpal. The graft was tensioned with the thumb held in approximately 40° of palmar abduction and neutral rotation, with the first metacarpal base reduced, using gentle manual tension sufficient to eliminate laxity without resisting passive abduction, and was secured to itself in that position using 3-0 braided polyester non-absorbable suture. This construct supplements the suspensory effect of the suture-button device and reflects principles of ligament reconstruction described in prior literature [5,7,8,11].

**Figure 5:**
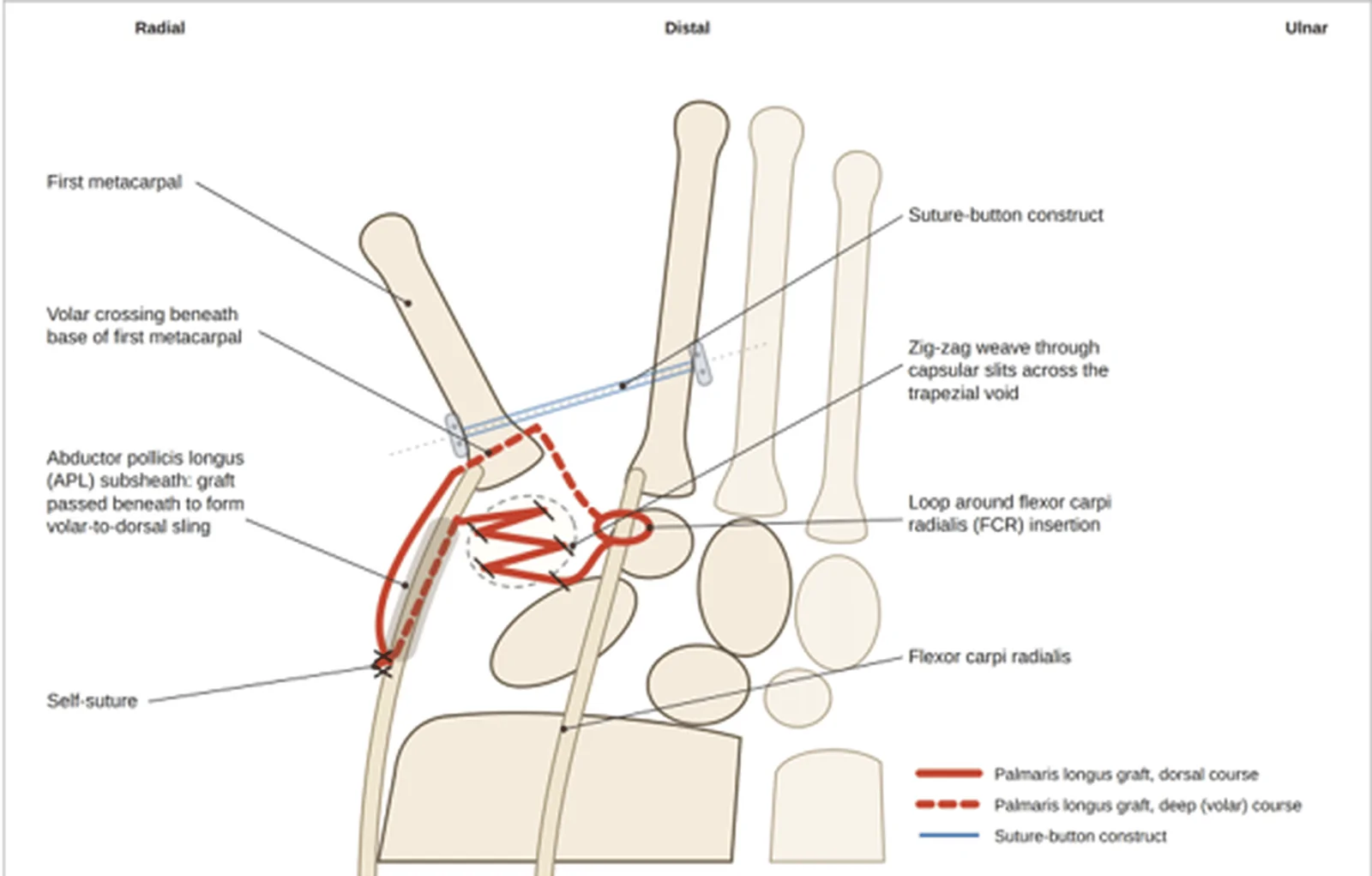
Schematic of the palmaris longus zig-zag reconstruction. Schematic of the palmaris longus zig-zag reconstruction, showing the graft passing beneath the abductor pollicis longus subsheath to create the volar-to-dorsal sling, the zig-zag weave through the capsular slits, the loop around the flexor carpi radialis insertion, the volar crossing beneath the base of the first metacarpal, and the final self-suture, drawn in the same plane as the suture-button construct. The schematic line diagram was drawn as a vector illustration with the assistance of Claude (Anthropic, San Francisco, USA). The authors then reviewed and corrected it for anatomical and technical accuracy.

The construct requires approximately 8-10 cm of tendon: the trapezial void after complete trapeziectomy measures roughly 1.5-2 cm, the run beneath the abductor pollicis longus subsheath and around the flexor carpi radialis insertion accounts for a further 4-5 cm, and the remaining length is used for the zig-zag weave and the self-suture. A margin therefore remained in all patients, and in no case was an alternative or supplementary graft required.

The capsule was closed with interrupted 3-0 braided polyester non-absorbable sutures, followed by skin closure with 4-0 nylon interrupted sutures. Capsular repair was emphasised to enhance long-term stability [9].

Postoperative rehabilitation

All patients followed an identical written rehabilitation protocol supervised by a single hand therapist. The thumb was immobilised in a forearm-based thumb spica splint for 10 days. From day 10, following suture removal, active and active-assisted thumb range-of-motion exercises, opposition and web-space stretches were begun, with the splint retained between exercise sessions and at night until three weeks. Progressive grip and pinch strengthening was introduced between weeks 2 and 4. Unrestricted activity was permitted from six weeks as tolerated. No patient received a modified protocol, and adherence was recorded at each therapy visit.

The period of splintage is determined by skin and capsular healing and by patient comfort rather than by the need to protect the reconstruction, because the cortical button-to-button device suspends the first metacarpal from the second from the moment it is deployed. Immobilisation of 7 to 14 days is consistent with the published protocols of Yao and Song [15], Cox et al. [18], and Yao and Cheah [16].

## Results

All 10 patients completed the 2-month follow-up. No patient was lost to follow-up. Preoperative and postoperative comparisons demonstrated statistically significant improvements in pain, function, range of motion (ROM), and radiographic parameters (Table 1).

**Table 1:** Baseline demographic and clinical characteristics of the study participants.

Parameter	Value
Number of patients	10
Age (range)	50-75 years
Mean age	62 years
Gender	2 males, 8 females
Dominant hand right	7
Dominant hand left	3
Eaton-Littler stage III	6
Eaton-Littler stage IV	4
Diabetes mellitus	3
Hypertension	3
Rheumatoid arthritis	2
No comorbidities	2

Pain, grip strength, and pinch strength all improved significantly between the preoperative assessment and the two-month review. Median VAS fell from 8.1 to 2.2 (Z = −2.803, p = 0.005, r = 0.89). Median grip strength rose from 54% to 74% of the contralateral hand (Z = −2.701, p = 0.007, r = 0.85), an absolute gain of approximately 20%. Median pinch strength rose from 48% to 74% of the contralateral hand (Z = −2.701, p = 0.007, r = 0.85). Pinch strength at two months appears favourable compared with the early postoperative values reported in previously published ligament reconstruction and tendon interposition series [15]; no direct comparison was undertaken in the present study, and the observation should be interpreted accordingly. All three effect sizes were large (Table 2).

**Table 2:** Pain, grip strength, and pinch strength before surgery and at two months, with Wilcoxon signed-rank test statistics. VAS: visual analogue scale; IQR: interquartile range; Z: Wilcoxon signed-rank test statistic; r: effect size, calculated as Z divided by the square root of the number of observations. Z, p, and r refer to the preoperative versus two-month paired comparison. N = 10 for every comparison.

Outcome measure	Preoperative median (IQR) [mean ± SD]	2 months median (IQR) [mean ± SD]	Median change (IQR)	Z	p-value	r
VAS for pain	8.1 (8–9) [8.2 ± 0.8]	2.2 (1–3) [2.3 ± 0.9]	−6.0 (−7 to −5)	−2.803	0.005	0.89
Grip strength, % of contralateral hand	54 (50–58) [54.2 ± 5.1]	74 (70–78) [74.1 ± 5.6]	+20 (17–23)	−2.701	0.007	0.85
Pinch strength, % of contralateral hand	48 (44–52) [48.3 ± 4.8]	74 (70–77) [73.8 ± 5.2]	+26 (22–29)	−2.701	0.007	0.85

The Wilcoxon signed-rank test demonstrated statistically significant improvements in thumb range of motion, with palmar abduction showing a Z value of −2.701 (p = 0.007, r = 0.85) and radial abduction showing a Z value of −2.497 (p = 0.013, r = 0.79). Both measures of thumb abduction improved significantly at two months. As this was a single-arm series, the relative contributions of trapeziectomy itself, the suture-button construct, the palmaris longus augmentation, and the early mobilisation protocol cannot be separated, and no causal attribution is made (Table 3; Figure 6C-6E).

**Table 3:** Thumb range of motion before surgery and at two months, with Wilcoxon signed-rank test statistics. °: degrees; IQR: interquartile range; SD: standard deviation; Z: Wilcoxon signed-rank test statistic; r: effect size, calculated as Z divided by the square root of the number of participants contributing paired observations. Z, p, and r refer to the preoperative versus two-month paired comparison. N = 10 for every comparison.

Variable	Preoperative median (IQR) [mean ± SD]	2 months median (IQR) [mean ± SD]	Median change (IQR)	Z	p-value	r
Palmar abduction (°)	35 (32–38) [35.2 ± 3.4]	57 (54–60) [56.8 ± 3.9]	+22 (19–24)	−2.701	0.007	0.85
Radial abduction (°)	32 (29–35) [32.1 ± 3.1]	47 (44–50) [47.3 ± 3.6]	+15 (12–17)	−2.497	0.013	0.79

**Figure 6:**
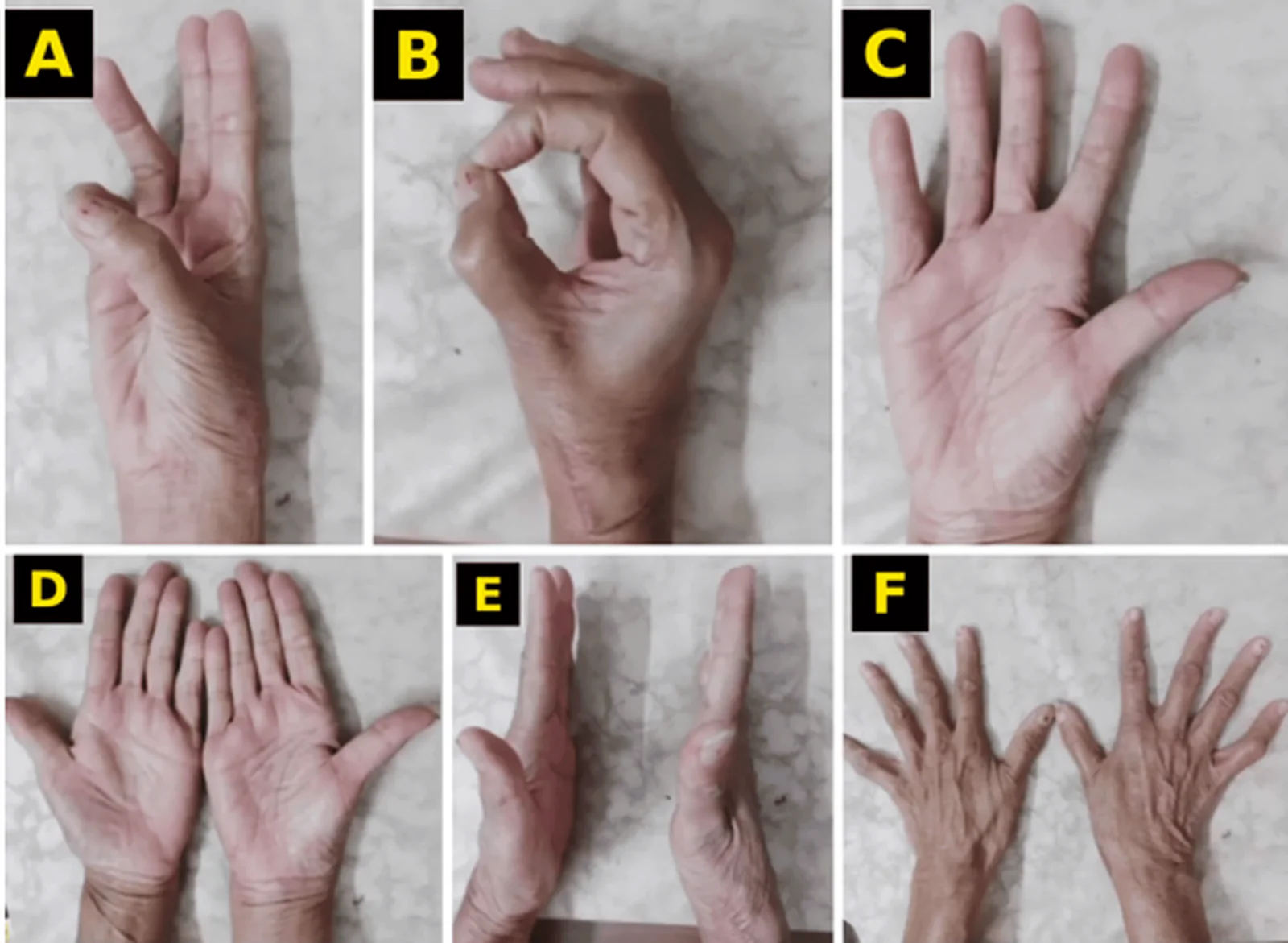
Postoperative clinical photographs of the operated hand at two months. (A) Thumb opposition to the base of the little finger. (B) Tip-to-tip pinch between the thumb and index finger. (C) Palmar abduction of the operated thumb. (D) Bilateral palmar view comparing palmar abduction of the operated and contralateral thumbs. (E) Bilateral view comparing radial abduction. (F) Bilateral dorsal view showing symmetrical thumb position and a well-healed incision. Written consent for publication was obtained from the patient shown.

The mean postoperative trapezial space ratio was 84% of the immediate postoperative value, with no radiographic evidence of button migration or metacarpal subsidence beyond this level (Table 4; Figure 5A, 5B). The trapezial space ratio in the present series was stable at two months. Whether this reflects the suspensory construct, the volar augmentation, or simply the short interval since surgery cannot be determined in the absence of a comparison group and longer follow-up.

**Table 4:** Radiological outcome - trapezial space ratio at two months. TSR: trapezial space ratio, defined as the distance from the base of the first metacarpal to the distal pole of the scaphoid divided by the length of the proximal phalanx of the thumb, expressed as a percentage of the immediate postoperative value. ICC: intraclass correlation coefficient; CI: confidence interval. The trapezial space ratio has no preoperative comparator because it is expressed relative to the immediate postoperative value; no effect size is therefore reported.

Parameter	Value	Statistical test
Mean postoperative TSR (% of immediate postoperative value)	84	Not applicable — single postoperative measurement
Interobserver agreement for TSR (ICC, 95% CI)	0.94 (0.85–0.98)	Two-way random-effects model, absolute agreement, single measures

The QuickDASH score was used to quantify functional improvement [22]. The Wilcoxon signed-rank test revealed a statistically significant and clinically meaningful improvement in function at two months (Z = −2.803, p = 0.005), with a large effect size (r = 0.89) (Table 5; Figure 6).

**Table 5:** QuickDASH functional scores before surgery and at two months, with Wilcoxon signed-rank test statistics. QuickDASH: shortened Disabilities of the Arm, Shoulder and Hand questionnaire; IQR: interquartile range; SD: standard deviation; Z: Wilcoxon signed-rank test statistic; r: effect size, calculated as Z divided by the square root of the number of participants contributing paired observations. The test statistics refer to the preoperative versus two-month paired comparison. N = 10.

Timepoint	Median QuickDASH (IQR)	Mean QuickDASH (± SD)	Comparison (preoperative vs 2 months)
Preoperative	58.0 (50.0–66.5)	58.4 ± 12.1	Z = −2.803; p = 0.005; r = 0.89
2 months postoperative	21.5 (15.9–27.3)	21.7 ± 8.5	
Median change (IQR)	−36.5 (−43.0 to −30.0)	−36.7 ± 9.8	—

There were no postoperative infections, hardware failures, or suture-button cut-outs. One patient developed a superficial radial nerve neurapraxia, which resolved completely within three weeks, and one patient experienced a transient complex regional pain syndrome, which resolved completely with conservative management. No patient developed persistent complex regional pain syndrome.

All outcome measures demonstrated statistically significant improvement, with large effect sizes ranging from 0.79 to 0.89, indicating a substantial clinical impact. Radiographic evaluation confirmed stable metacarpal suspension, and no major complications were observed. The QuickDASH score improved by 62.8%, reflecting rapid restoration of functional capacity consistent with early rehabilitation protocols following suture-button suspensionplasty (Table 6). Effect sizes for every outcome are reported in Table 6: VAS for pain r = 0.89; grip strength r = 0.85; pinch strength r = 0.85; palmar abduction r = 0.85; radial abduction r = 0.79; QuickDASH r = 0.89.

**Table 6:** Summary of all outcome measures with Wilcoxon signed-rank test statistics. VAS: visual analogue scale; QuickDASH: shortened Disabilities of the Arm, Shoulder and Hand questionnaire; TSR: trapezial space ratio; °: degrees; Z: Wilcoxon signed-rank test statistic; r: effect size, calculated as Z divided by the square root of the number of participants contributing paired observations. Z, p, and r refer to the preoperative versus two-month paired comparison. N = 10 for every comparison. The trapezial space ratio has no preoperative comparator and is therefore reported without test statistics.

Outcome	Preoperative	Postoperative	Z	p-value	Effect size (r)
VAS for pain	8.1	2.2	−2.803	0.005	0.89
Grip strength (%)	54	74	−2.701	0.007	0.85
Pinch strength (%)	48	74	−2.701	0.007	0.85
Palmar abduction (°)	35	57	−2.701	0.007	0.85
Radial abduction (°)	32	47	−2.497	0.013	0.79
QuickDASH	58.4	21.7	−2.803	0.005	0.89
TSR (%)	—	84	—	—	—

## Discussion

In this small, single-arm prospective case series, trapeziectomy with suture-button suspensionplasty combined with a palmaris longus zig-zag augmentation was associated with improvements in pain, strength, range of motion, and radiographic trapezial height at two months. The magnitude of the within-subject changes was large, but in the absence of a comparison group, these findings are descriptive and hypothesis-generating rather than confirmatory.

These findings are consistent with existing SBS literature reporting rapid rehabilitation, durable maintenance of metacarpal height, and low complication rates [14-20]. The objective of describing early clinical, functional and radiographic outcomes following suture-button suspensionplasty augmented with a palmaris longus construct was met. The results are consistent with, but do not establish, a contribution from combined suspensory and volar stabilisation; the study was not designed to test mechanism or to demonstrate superiority over any alternative procedure.

One of the major advantages of suture-button suspensionplasty (SBS) is the ability to permit early mobilisation. Yao and Song demonstrated that stable suspensory fixation allows initiation of motion within the first postoperative weeks, reducing stiffness and facilitating faster recovery compared with traditional ligament reconstruction and tendon interposition (LRTI) procedures [15]. Earlier rehabilitation has consistently been associated with improved patient satisfaction and more rapid return to activities of daily living.

While simple trapeziectomy reliably alleviates pain, it does not prevent proximal migration of the first metacarpal [13]. The postoperative trapezial space ratio (TSR) of 84% observed in this study aligns with prior reports by DeGeorge et al. and Yao and Cheah [14,16,18]. These radiographic findings parallel the early gains in grip strength, pinch strength, and thumb motion observed in our cohort.

Ligament reconstruction and tendon interposition (LRTI), as described by Froimson and popularised by Burton and Pellegrini, remains widely practised but is associated with donor-site morbidity, increased soft-tissue dissection, and prolonged rehabilitation [7,8]. Moreover, comparative studies have failed to demonstrate clear superiority of LRTI over trapeziectomy alone [12]. In contrast, SBS avoids tendon harvest, limits surgical morbidity, and facilitates early mobilisation. Early strength recovery at two months in the present series appears favourable compared with the trajectories reported following LRTI; no direct comparison was undertaken here. In a cadaveric loading model, suture-button constructs resisted metacarpal subsidence significantly better than LRTI at every increment of applied load [23]. We hypothesise that this greater resistance limits metacarpal settling during the early healing period and thereby contributes to the rapid recovery of grip and pinch strength observed in the present series. This mechanism was not tested here, and the hypothesis would require a comparative study with serial radiographic and strength measurements to examine.

Soft-tissue suture suspension arthroplasty (SSA) has demonstrated wide variability in radiographic subsidence, averaging 35% and ranging from 0% to 90% at 12-to 14-year follow-up [11]. Several factors plausibly account for this variability. Soft-tissue suture suspension arthroplasty relies on a suture tether between the bases of the first and second metacarpals without cortical button fixation, so load is borne by suture-bone interfaces that may cut out or elongate to differing degrees. Suture material, number of passes and tensioning are not standardised between surgeons. Radiographic measurement of trapezial height is itself inconsistent across the literature, with several indices in use and no agreed reference, which widens the reported range independently of the biological result. Finally, subsidence continues until a stable fibrous pseudarthrosis forms, so the degree recorded depends heavily on the interval at which the radiograph is taken, and the cited series reports 12- to 14-year follow-up [11]. Importantly, this radiographic variability has not translated into a comparable variability in reported clinical outcome.

The biomechanical rationale for PL zig-zag augmentation is based on restoration of the volar beak ligament, a primary stabiliser resisting dorsal subluxation of the first CMC joint [5]. Classical reconstructive procedures similarly recreate this volar restraint using tendon grafts [7,8]. The proposed mechanism is that this combined stabilisation reduces early micromotion and reinforces the capsular envelope, which would be consistent with the early range-of-motion gains, the maintained trapezial height, and the absence of early subsidence observed here. This mechanism is inferred from the construct’s design and from cadaveric data; it was not directly demonstrated in the present study. No PL harvest-related complications were recorded.

Previous comparative studies have demonstrated shorter operative times with SBS compared with LRTI. Yao and Cheah reported reductions in surgical duration and faster return to unrestricted activity, making SBS particularly attractive in elderly patients and individuals desiring early functional recovery [16].

Complications specific to SBS described in the literature include second metacarpal fractures, symptomatic implant prominence, index metacarpal pain, button pullout, and extensor tendon irritation [17,20]. These complications are uncommon and are largely technique-dependent. Careful positioning of the second metacarpal tunnel, avoidance of excessive tensioning, and fluoroscopic confirmation of implant placement are considered important technical pearls that minimise these risks. None of these complications occurred in the present series. There were no second metacarpal fractures, no button pull-out or migration, no symptomatic implant prominence, no index metacarpal pain, and no extensor tendon irritation at two months. The two adverse events recorded were a transient complex regional pain syndrome and a superficial radial nerve neurapraxia, both of which resolved completely with conservative management; the latter is attributable to the dorsoradial approach and reinforces the importance of careful protection of the superficial radial nerve branches. The follow-up interval must be borne in mind, however, since symptomatic implant prominence, second metacarpal stress fracture and late button-related problems are typically reported beyond two months, and their absence here cannot be taken as evidence that they will not occur.

Long-term investigations of LRTI and trapeziectomy have consistently shown that pain relief is reliable irrespective of technique, whereas differences between procedures are more apparent during the early postoperative period. Systematic reviews by Vermeulen et al. and Wajon et al. concluded that no single procedure has demonstrated clear superiority in long-term pain outcomes [12,24]. Consequently, the principal advantage of SBS appears to lie in accelerated recovery, earlier return of strength, preservation of metacarpal height, and shorter rehabilitation periods rather than superior long-term pain relief.

Because bone stock and the surrounding soft tissues are relatively preserved, revision options are reported to remain available following suture-button suspensionplasty [14,16], which may be of relevance in younger or higher-demand patients. Revision surgery was not required or assessed in the present series, and this consideration is therefore drawn from the published literature rather than from our own data.

The QuickDASH score in the present series improved from 58.4 ± 12.1 preoperatively to 21.7 ± 8.5 at two months, a reduction of 62.8% (p = 0.005, r = 0.89). This is of a similar order to the functional improvement reported in previous suture-button suspensionplasty cohorts [15,17,20]; those series, however, used differing instruments and follow-up intervals ranging from two to more than ten years, so the comparison is indicative rather than direct.

Strengths

Key strengths include the prospective design, a standardised surgical and rehabilitation protocol applied to all patients, combined radiographic and functional assessment, outcome assessment by an assessor who was not the operating surgeon, appropriate non-parametric statistical analysis, and a step-by-step description of the palmaris longus zig-zag augmentation supported by schematic diagrams and by intraoperative photographs of the suture-button steps.

Limitations and future directions

The principal limitation of this study is the absence of a control group. Because all patients received the same procedure, the observed improvements cannot be attributed to the palmaris longus augmentation, to the suture-button construct, to the trapeziectomy itself or to the early mobilisation protocol, and no comparison can be made with trapeziectomy alone, with ligament reconstruction and tendon interposition or with standalone suture-button suspensionplasty. The small sample size and the short two-month follow-up compound this, and together they confine the study to a descriptive, hypothesis-generating role.

Further limitations include the absence of radiographic follow-up beyond two months, which means that progressive subsidence, late loss of trapezial height, button migration and implant-related complications cannot be excluded; the absence of any generic or condition-specific quality-of-life instrument, such as the EQ-5D, SF-36 or Michigan Hand Outcomes Questionnaire, alongside QuickDASH, together with the absence of a patient satisfaction measure, return-to-work data and a formal pain-medication record; the absence of preoperative clinical photographs, so that the preoperative deformity is documented radiographically (Figure 1) but not photographically; the absence of blinding of the assessor to the timepoint; the single-surgeon, single-centre design, which means that reproducibility across surgeons and the learning curve were not assessed; the clinical and fluoroscopic rather than instrumented assessment of graft and construct tension, which introduces surgeon-dependent variability; and the expression of the trapezial space ratio relative to the immediate postoperative value rather than to a preoperative baseline, which permits a statement about maintenance of trapezial height but not about its restoration.

Future work should take the form of a randomised comparison of suture-button suspensionplasty with and without palmaris longus augmentation, adequately powered on the effect sizes observed here, with follow-up of at least 12 months, serial radiographic assessment of the trapezial space ratio, inclusion of quality-of-life and satisfaction instruments, and formal assessment of reproducibility across multiple surgeons including the learning curve, before the technique could be recommended for general adoption.

## Conclusions

In this pilot prospective case series of 10 patients, trapeziectomy with suture-button suspensionplasty combined with palmaris longus zig-zag augmentation was associated with reduced pain, improved grip and pinch strength, improved thumb range of motion and maintained trapezial height at two months, with no infections, hardware failures or button cut-outs and with two adverse events that resolved completely. Because the series was single-armed, small, and assessed at two months only, these findings are descriptive and hypothesis-generating: they do not establish superiority over any alternative procedure, do not isolate the contribution of the palmaris longus augmentation, and do not address durability of the clinical result or late implant-related complications. A randomised comparison with longer follow-up is required before the augmentation can be recommended for general adoption.
